# Identification of predictive factors for outcomes after robot-assisted partial nephrectomy based on three-dimensional reconstruction of preoperative enhanced computerized tomography

**DOI:** 10.3389/fonc.2023.927582

**Published:** 2023-02-28

**Authors:** Qinyu Li, Yucong Zhang, Man Liu, Heng Li, Wei Guan, Xiaoyan Meng, Zhiquan Hu, Zhihua Wang, Shaogang Wang, Zhen Li, Jihong Liu, Zheng Liu

**Affiliations:** ^1^ Department of Urology, Tongji Hospital, Tongji Medical College, Huazhong University of Science and Technology, Wuhan, China; ^2^ Department of Geriatrics, Tongji Hospital, Tongji Medical College, Huazhong University of Science and Technology, Wuhan, China; ^3^ Department of Radiology, Tongji Hospital, Tongji Medical College, Huazhong University of Science and Technology, Wuhan, China

**Keywords:** tumor bed area, kidney cancer, nomogram, warm ischemia time, robot-assisted partial nephrectomy

## Abstract

**Background:**

Information from the RENAL score is limited. This study aimed to identify new parameters based on three-dimensional (3D) reconstruction of preoperative enhanced computerized tomography (CT) for predicting outcomes after robot-assisted partial nephrectomy (RPN).

**Materials and methods:**

The records of kidney cancer patients who underwent RPN at Tongji Hospital from March 2015 to July 2019 were reviewed. Demographic data, laboratory examinations, postoperative hospitalization time, and enhanced CT were retrospectively collected. Some tumor parameters were obtained from 3D reconstruction of CT data. The association between these predictive factors and outcomes after RPN was analyzed.

**Results:**

A larger tumor bed area (TBA) was associated with a longer warm ischemia time (WIT) (P-value <0.001) and tumor resection time (P-value <0.001). Moreover, TBA was significantly associated with the elevation of postoperative creatinine (P-value = 0.005). TBA (P = 0.008), distance from the tumor to the first bifurcation of the renal artery (DTA) (P <0.034), and RENAL score (P = 0.005) were significantly associated with WIT in univariate logistic regression. In multivariate logistic regression, TBA (P = 0.026) and DTA (P = 0.048) were independent risk factors for prolonged WIT (over 25 min). The predictive effect of the combination of TBA, DTA, and RENAL score was higher than the predictive effect of RENAL score alone for WIT (area under curve: 0.786 versus 0.72).

**Conclusion:**

TBA and DTA are independently associated with the WIT of RPN, which provides additional assessment value for the complexity of kidney cancer in RPN over the RENAL score.

## Introduction

Malignant kidney tumors account for 2% of the global cancer burden. In 2018, 350,000 new cases occurred worldwide (1). Owing to improvements in imaging examination and timely diagnosis, the incidence of renal cell carcinoma (RCC) has increased in recent years (2). Accordingly, the incidental detection of renal masses is also rising (3). Currently, advances in surgical techniques have made partial nephrectomy (PN) the preferred treatment option for kidney cancers that are feasible to resect. In fact, PN is recommended for small renal masses by the guidelines (4), and its indication has extended from cT1a (diameter ≤4 cm) to some cT1b (4 cm <diameter ≤7 cm) (5) and even cT2a (7 cm <diameter ≤10 cm) renal tumors (6).

Currently, PN can be performed by open conventional laparoscopic or robot-assisted laparoscopic methods (7, 8). The choice of surgical modality is based on multiple factors, such as the characteristics of the tumor and patient (9). Among them, robot-assisted PN (RPN) has become increasingly popular for cT1 tumors, especially in some complex conditions (10). RPN is attractive owing to the combination of minimal invasiveness and the flexibility of the surgeon’s hand, and recent evidence has suggested that RPN has advantages over open and conventional laparoscopic PN in terms of surgical outcomes and hospitalization time (11, 12).

It is obvious that renal function might decrease after PN. Extensive studies have shown that the amount of resected healthy parenchyma, ischemia time, and reconstructive injury are critical factors involved in the loss of renal function (13, 14). Although the quantity and quality of preserved parenchyma are the most important determinants of postoperative renal function, prolonged warm ischemia time (WIT) also plays a significant role in renal function recovery (15–17). Available data suggest a benefit of keeping WIT <25 min in PN, and prolonged WIT is significantly correlated with worse postoperative renal function (15).

Over the past decades, factors related to tumor anatomy have been unified in different scoring systems to predict the complexity of surgery (18–22). Among them, the RENAL score is widely used in clinical practice. This scoring system was proposed in 2009 and is based on five critical anatomical characteristics of solid renal masses (20). However, with the development and extensive application of RPN, some limits to the RENAL score have been observed. The main deficiency is that the components of the RENAL score are factors in a two-dimensional plane. Nevertheless, PN surgery is a three-dimensional (3D) process, especially in the RPN. Therefore, we believe that the RENAL scoring system should be updated considering the rapid advancement of new imaging features. Here, we identified a new parameter, the tumor bed area (TBA), which is the contact area between the tumor and adjacent normal tissues, and obtained several other new parameters from 3D reconstruction of enhanced abdominal computerized tomography (CT), including the ratio of TBA over the tumor surface area, the volume of the tumor mass, and the distance from the tumor to the first bifurcation of the renal artery (DTA). We evaluated the associations between these parameters and operative and postoperative indicators by logistic regression analysis and correlation analysis. Additionally, a new updated scoring system was illustrated in a predictive nomogram, which could help surgeons identify patients with high complexity who may suffer longer WIT during RPN.

## Materials and methods

### Patient selection

This study was approved by the Ethics Committee of Tongji Hospital (TJ-IRB20220407) and was carried out in accordance with the ethical standards of the Helsinki Declaration. The STROCSS criteria are provided as a supplementary file. This study was a cross-sectional study that retrospectively collected clinical data from kidney cancer patients who underwent RPN at Tongji Hospital from March 2015 to July 2019. The inclusion criteria for patients were as follows: (1) age ≥18 and ≤80; (2) the surgery record video was available; and (3) an enhanced abdominal CT was performed before surgery. The exclusion criteria were as follows: (1) renal failure (creatinine >707 μmol/L), (2) solitary kidney, and (3) anatomical abnormalities of the urological system.

### Procedure of RPN

An expert surgeon completed all the operations transperitoneally. Prior to general anesthesia, a Foley catheter was inserted. The patient was placed in a flank position at approximately 70°. Then, five trocars were inserted (three 12 mm trocars and two 8 mm trocars). Dissection of the renal capsule was accomplished after opening Gerota’s fascia. Around the potential tumor location, fat was removed circumferentially. The tumor boundary was defined with a laparoscopic ultrasonography probe in the case of an endophytic kidney tumor. After clamping the renal artery, the surgeon cut the border with cold scissors. The tumor was dissected along with the pseudocapsule until the tumor’s bottom was reached. The tumor was then pulled upward and dissected to the left, right, and forward until it reached the renal capsule. Renorrhaphy was conducted on two layers of the kidney: the renal medulla and the parenchyma. Sutures were also placed in the renal medulla and parenchyma, as well as obvious tears in blood vessels and collecting systems, while suturing the renal medulla. After unclamping the renal artery, the renal excision bed was examined for hemostasis. In the end, a drainage tube was inserted. The Foley catheter remained in place.

### Data collection

Demographic data (age and sex), laboratory examinations (leukocytes in blood, hemoglobin, creatinine, and eGFR), and postoperative hospitalization time were retrospectively recorded. Moreover, enhanced abdominal CT data were obtained for 3D reconstruction, and surgery record video was also obtained for assessment of operative time. Finally, we calculated the operation time, including WIT, tumor resection time, and suturing time, by artificially scanning the surgery record video. In detail, the surgery video was recorded through the camera of the Intuitive Surgical DaVinci S/Si system. The time from clipping the renal artery (**Figure S1A**) to reperfusion (**Figure S1B**) was defined as WIT. Tumor resection time was defined as the time from the incision of the renal parenchyma by an ultrasonic scalpel (**Figure S1C**) to the complete separation of the tumor mass (**Figure S1D**), and suturing time was defined as the time of suturing the tumor bed to the renal parenchyma (**Figures S1E, F**).

### Calculation of indicators based on preoperative enhanced abdominal CT

**Figure 1:**
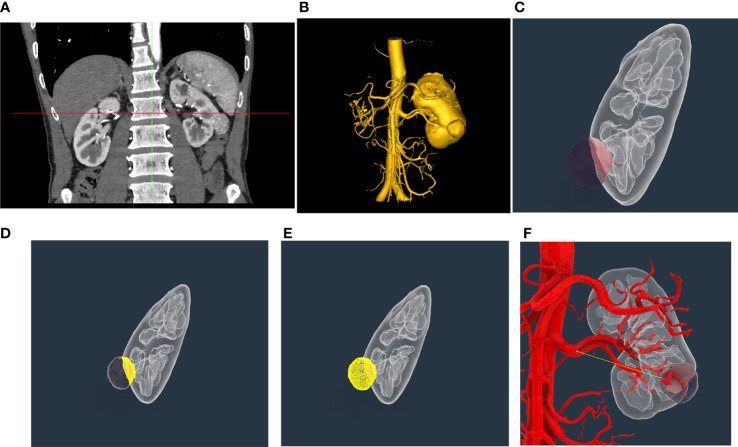
Representative three-dimensional (3D) reconstruction of enhanced abdominal CT and measurement of indicators of tumor mass. **(A)** Representative enhanced abdominal CT scan image of a kidney cancer patient. **(B)** Three-dimensional reconstruction of the kidney based on enhanced abdominal CT by Mimics 24. **(C)** Exhibition of tumor mass in Rhinoceros 3D; **(D)** Automatic calculation of tumor bed area using Rhinoceros 3D. **(E)** Automatic calculation of surface area and volume of tumor mass using Rhinoceros 3D. **(F)** Manual measure of the distance from the tumor to the first bifurcation of the renal artery in the tumor tissue using Rhinoceros 3D.

Enhanced abdominal CT data (**Figure 1A**) were imported into Mimics 24 (Materialise, Leuven, Belgium), which can automatically conduct 3D reconstruction (**Figure 1B**). Owing to the significant difference in absorption between tumor tissue and adjacent normal tissue in enhanced abdominal CT, Mimics 24 can also automatically distinguish tumor tissue from adjacent normal tissue. The 3D reconstruction model was then imported into Rhinoceros 3D (Rhino, Robert McNeel & Associates, Washington DC, USA) (**Figure 1C**), which can automatically calculate TBA (the contact area between tumor tissue and adjacent normal tissue) (**Figure 1D**) and the surface area and volume of the tumor mass (**Figure 1E**). The area ratio was defined as the ratio of TBA to the total surface area of the tumor mass. The distance from the tumor to the first bifurcation of the renal artery (DTA) was measured manually in Rhinoceros 3D (**Figure 1F**). The RENAL score was assessed by two experienced radiologists based on enhanced abdominal CT.

### Statistical analysis

All analyses were performed in RStudio 4.0.4. All correlation analyses were performed using the Spearman method. The cutoff values of the continuous variables, including TBA, ratio, volume, and distance, were obtained from the receiver operating characteristic (ROC) curve for WIT. Student’s t test and the Mann−Whitney U test were applied for two-group comparisons of normally or skewed distribution data, and a P-value <0.05 was regarded as statistically significant. Univariate logistic regression analysis was also executed to explore the associations between TBA, area ratio, volume, DTA, RENAL score, and WIT. Before multivariate logistic regression, we converted those continuous variables into binary variables according to the ROC curve, and significant variables (P-value <0.05) in univariate logistic regression were selected to perform multivariate logistic regression. The regression coefficients were proportionally converted to a scale of 0–100 points to generate a predictive nomogram. We assessed the efficiency of the model by calculating the area under the ROC curve (AUC).

## Results

**Figure 2:**
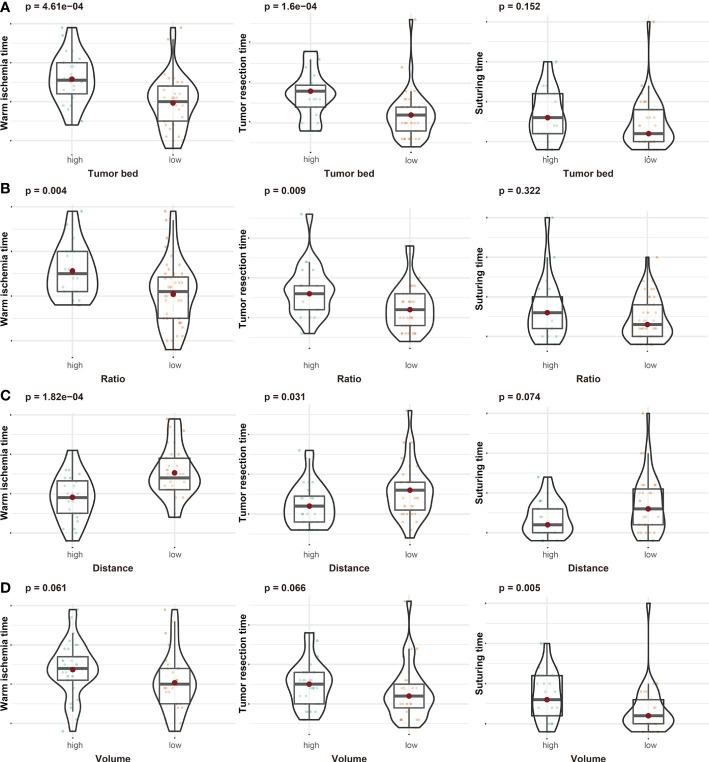
The associations between tumor bed area **(A)**, area ratio **(B)**, distance from the tumor to the first bifurcation of the renal artery **(C)**, tumor volume **(D)**, and operation time (including warm ischemic time, tumor resection time, and suturing time).

As shown in **Table 1**, data from 65 patients were included in this study. The RENAL score of these patients ranged from 5 to 11. A total of 22 patients had prolonged WIT (over 25 min) (**Table S1**). According to ROC curves (**Figure S2**), the thresholds for TBA, area ratio, DTA, and tumor mass volume were 0.269 dm^2^, 54.8%, 0.344 dm, and 0.023 dm^3^, respectively. We divided patients into different subgroups based on the thresholds and compared the operation time between the high- and low-risk groups. Patients with higher TBA had a longer WIT (P-value <0.001) and tumor resection time (P-value <0.001) (**Figure 2A**). Moreover, the WIT and tumor resection time of patients were positively associated with the area ratio (P-value = 0.004, P-value = 0.009, respectively) and negatively associated with DTA (P-value <0.001, P-value = 0.031, respectively) (**Figures 2B, C**). Interestingly, while the volume of the tumor was not associated with WIT or tumor resection time, it was significantly positively correlated with suturing time (**Figure 2D**).

**Table 1:** Demographic and clinical features in this study.

Characteristic	levels	Overall (n=65)
Age, years (mean ± SD)		50.00 ± 11.22
Gender, n (%)	Female	22 (34%)
	Male	43 (66%)
Position, n (%)	Left	28 (43%)
	Right	37 (57%)
RENAL Score, n (%)	5	8 (12%)
	6	10 (15%)
	7	15 (23%)
	8	10 (15%)
	9	8 (12%)
	10	10 (15%)
	11	1 (2%)
Tumor bed area, dm^2^ median (interquartile range)		0.20 (0.12 to 0.33)
leukocytes, 10^9/L (mean ± SD)		5.94 ± 1.42
Hb, g/L (mean ± SD)		138.00 ± 14.11
EGFR, ml/min (mean ± SD)		96.00 ± 16.70
Creatinine, umol/L median (interquartile range)		70.50 (64.75 to 83.25)
Postoperative stay, days median (interquartile range)		8 (7 to 9)

SD, Standard Deviation; Hb, Hemoglobin; EGFR, Estimated glomerular filtration rate.

**Figure 3:**
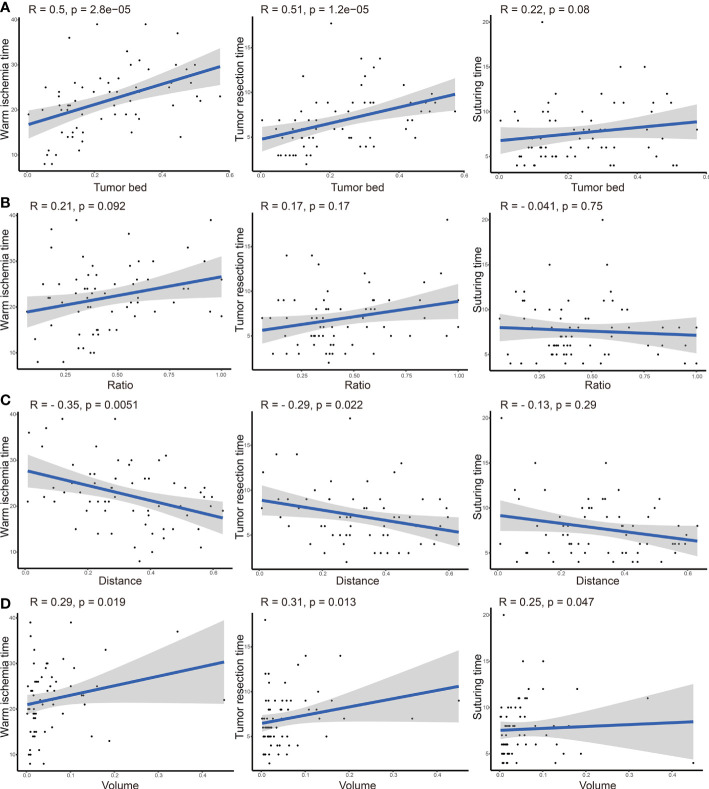
Correlation analysis between tumor bed area **(A)**, area ratio **(B)**, distance from the tumor to the first bifurcation of the renal artery **(C)**, tumor volume **(D)**, and operation time (including warm ischemic time, tumor resection time, and suturing time).

Additionally, we checked this assumption using a scatterplot. As displayed in **Figure 3**, TBA was significantly correlated with WIT (r = 0.5, P-value <0.001) and tumor resection time (r = 0.51, P-value <0.001). Moreover, the distance was significantly negatively correlated with the WIT (r = −0.35, P-value = 0.005), and the volume showed a positive correlation with the WIT (r = 0.29, P-value = 0.019), tumor resection time (r = 0.31, P-value = 0.013), and suturing time (r = 0.25, P-value = 0.047).

We also assessed the association between these variables and postoperative clinical parameters, including the change in leukocytes, hemoglobin, creatinine, eGFR, and postoperative stay (**Table 2**). TBA was significantly associated with the elevation of creatinine (P-value = 0.005) and the decline in eGFR (P-value = 0.004). Additionally, the volume of the tumor was remarkably associated with a decrease in hemoglobin. We also compared these clinical parameters between the high- and low-TBA, area ratio, tumor volume, and DTA groups (**Figures S3–6**). The results indicated that patients with a higher area ratio showed a higher elevation of creatinine (**Figure S4**), and patients with a larger tumor volume had more hemoglobin loss and longer hospitalization days after surgery (**Figure S6**).

**Table 2:** Correlations between risk factors and clinical parameters.

Variables	Leukocyte change		Hb lose		Creatinine change		EGFR lose		Postoperative stay	
	Cor	P	Cor	P	Cor	P	Cor	P	Cor	P
Tumor bed area	-0.081	0.540	0.230	0.074	0.360	**0.005**	0.270	**0.040**	0.250	0.059
Ratio	0.075	0.570	-0.170	0.180	0.220	0.097	0.180	0.170	-0.033	0.800
distance	-0.051	0.710	-0.086	0.520	-0.240	0.071	-0.140	0.300	-0.230	0.084
Volume	-0.033	0.800	0.280	**0.032**	0.210	0.110	0.170	0.200	0.240	0.070

P values less than 0.05 are bolded.

**Figure 4:**
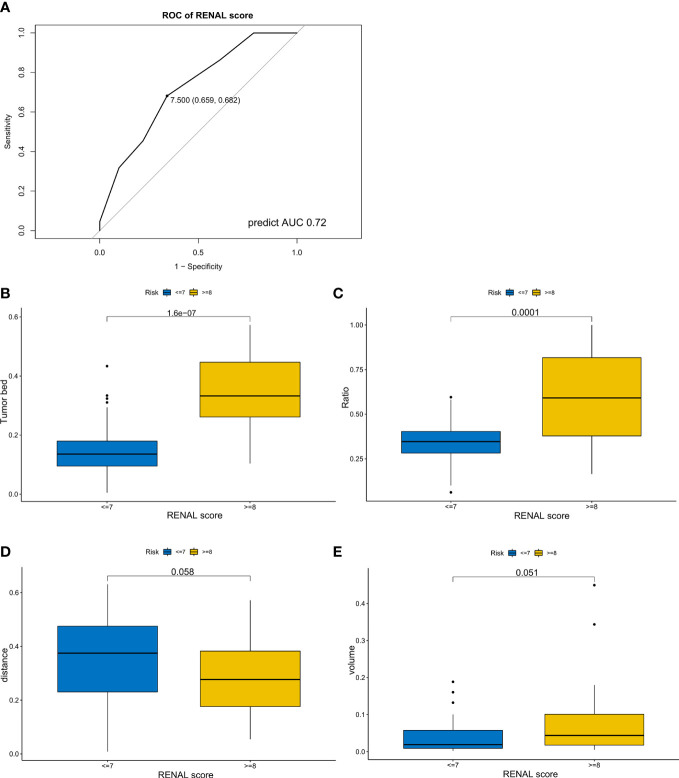
The associations between tumor features and RENAL score. **(A)** Receiver operating characteristic curve of RENAL score for longer warm ischemic time (over 25 min). **(B–E)** Differences in tumor bed area **(B)**, area ratio **(C)**, distance from the tumor to the first bifurcation of the renal artery **(D)**, and tumor volume **(E)** between the high and low RENAL score subgroups.

Considering the importance of the RENAL score in the preoperative evaluation of kidney cancer, we explored the associations between these four variables and the RENAL score. The ROC curve was applied to determine the threshold of the RENAL score (**Figure 4A**), and we compared the TBA, area ratio, tumor volume, and DTA between the high and low RENAL score groups. Patients with higher TBA (P-value <0.001) and area ratio (P-value <0.0001) showed higher RENAL scores (**Figures 4B, C**). Although statistically insignificant, patients with higher DTA (P-value = 0.058) and tumor volume (P-value = 0.051) may also have higher RENAL scores (**Figures 4D, E**).

Then, we performed logistic regression analysis to further validate the associations between the four variables, RENAL score, and WIT. In univariate logistic regression analysis, TBA (OR = 237.679, P = 0.008), DTA (OR = 0.021, P <0.034), and RENAL score (OR = 1.660, P = 0.005) were significantly associated with the WIT of patients (**Table 3**). In multivariate logistic regression analysis, TBA (OR = 4.475, P = 0.026) and DTA (OR =0.273, P = 0.048) were independent risk factors for prolonged WIT (over 25 min) (**Table 3**). We also assessed the multicollinearity of the TBA, DTA, and RENAL scores. The variance inflation factor indicated that multicollinearity was not a concern (variance inflation factor <2) (**Table S2**).

**Table 3:** Risk factors for longer warm ischemia time (over 25 mins).

Variable	univariate logistics regression			multivariate logistics regression		
	OR	95% CI	P Value	OR	95% CI	P Value
Tumor bed area	237.679	4.046-13962.742	0.008^*^	4.475	1.193-16.790	0.026^*^
Ratio	4.351	0.464-40.814	0.198	NA	NA	NA
Distance	0.021	0.001-0.750	0.034^*^	0.273	0.075-0.990	0.048^*^
Volume	26.730	0.034-20822.470	0.333	NA	NA	NA
RENAL score	1.660	1.166-2.363	0.005^*^	1.564	0.408-5.996	0.514

NA, not applicable.

*P value < 0.05.

P values less than 0.05 are bolded.

**Figure 5:**
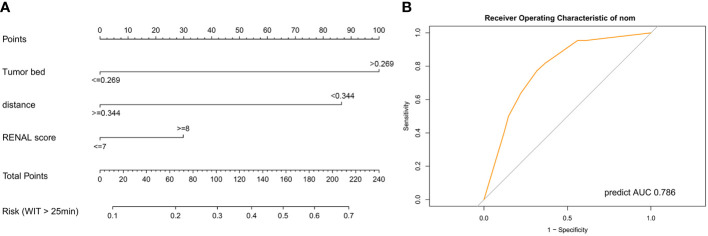
Predictive model for warm ischemic time of patients in robot-assisted partial nephrectomy. **(A)** Nomogram model for the risk of prolonged warm ischemic time (over 25 min). **(B)** Receiver operating characteristic curve of the nomogram model. AUC, area under curve.

For better application in clinical practice, we constructed a nomogram model based on the results of multivariate logistic regression (**Figure 5A**). A score was assigned to each factor according to the point scale, and the total score was obtained by adding the assigned score. The corresponding risk for prolonged WIT (over 25 min) was illustrated by the total points axis. The Hosmer–Lemeshow test was conducted to assess whether the difference between the predicted value and the actual data was significant, and the final model showed a good fit (P-value = 0.998) (**Figure S7**). Finally, we evaluated the efficiency of the model using the ROC curve, and the AUC was 0.786 (**Figure 5B**), which was greater than the prediction by the RENAL score alone (AUC = 0.72) (**Figure 4A**).

## Discussion

PN is widely accepted as the first treatment choice for small renal masses (4). With the increasing accessibility of robotics, an increasing number of surgeons prefer to perform RPN (23) as it can achieve the same effect of tumor control as radical nephrectomy and offers better preservation of renal function (24). Currently, the indications for RPN are expanding (6), which means a higher possibility of tackling complex and difficult tumors. Apart from preserving nephrons, WIT is considered to play a critical role in postoperative renal function (25–27). Several studies have revealed that prolonged WIT (over 25 min) could cause long-lasting diffuse damage to the operated kidney (26, 28). Therefore, prediction of the possibility of a longer WIT is critical and may help surgeons make treatment decisions.

The RENAL score is a widely used standardized nephrometry scoring system that can quantify the anatomical features of renal masses based on imaging examinations (29, 30). The RENAL score is supposed to indicate the intricacy of the procedure. A total score of 4–6 is regarded as low complexity, 7–9 is regarded as intermediate complexity, and beyond 10 is regarded as high complexity (20). Numerous studies have demonstrated a substantial association between the RENAL score and perioperative outcomes and complications (31–34). The RENAL score can be used as a reference for the selection of a surgical approach. OPN is suited to complex tumors, while conventional laparoscopic PN and RPN favor patients with lower RENAL scores (32). Moreover, a higher RENAL score was associated with several postoperative complications, such as pelvicalyceal entry, urine leakage, increased blood loss, WIT, and length of hospital stay. However, with the development of robot-assisted technology, some limitations of the RENAL score appeared. In this study, we identified some new parameters based on 3D reconstruction of enhanced abdominal CT. Considering the highly irregular shape and infiltrative growth pattern of the tumor, we believed these parameters could better reflect the anatomical features of the tumor in 3D. To verify this conjecture, we collected clinical information, operation time, and enhanced CT data from 65 patients.

According to the results, patients with high TBA had a longer WIT and tumor resection time in the RPN. Strong correlations between WIT, tumor resection time, and TBA were observed. Moreover, TBA was significantly related to less renal function loss. Additionally, it was found that patients with higher TBA showed a higher RENAL score. All the results suggested that TBA may be used as a predictive marker for outcomes after RPN. In the RENAL score, maximal diameter is an indicator to characterize anatomical attributes and evaluate the resectability of renal tumors (20). However, in the actual scenario, the contact area has a greater effect on surgical difficulty than the maximal diameter. Even after incorporating endophytic properties, tumors with the same RENAL score still show different difficulty levels. The RENAL score only categorizes tumors into nine grades (4 to 12), while TBA can reflect surgical difficulty quantitatively and precisely. Additionally, compared with manual measurement of the RENAL score, TBA could be obtained automatically from the software, which also reduces potential bias.

Several other new parameters obtained from 3D reconstruction of enhanced CT were also included in this analysis, including the area ratio, tumor volume, and DTA. The area ratio quantitatively assesses the endophytic properties of the tumor. The DTA quantitatively reflects the risk of vascular injuries during surgery. Interestingly, DTA was also an independent risk factor for prolonged WIT (over 25 min) in multivariate logistic regression.

We also established a nomogram model based on the outcomes of multivariate logistic regression (35). TBA, DTA, and RENAL scores were included in this scoring system. As shown in the nomogram, TBA accounted for the highest proportion of the risk of prolonged WIT, followed by DTA. TBA and DTA showed potential advantages for predicting the WIT of patients over the RENAL score. The Hosmer–Lemeshow test (36) suggested a limited difference between the predicted value and the actual data of this model (P-value = 0.998). Furthermore, the AUC of the nomogram was 0.786, which was higher than the prediction by the RENAL score alone (AUC = 0.72). All these results indicated the potential of TBA in predicting the WIT of patients during RPN.

However, several limitations also exist. First, this study was a retrospective analysis with a limited sample size. In the absence of a validation cohort, the findings remain speculative. Therefore, prospective studies with large samples are needed to validate the results. Second, the DTA was measured manually, which might bring in small errors. Third, the spatial distribution and location of the tumor are very important and have a significant impact on the difficulty of surgery. We will try to include this feature in the scoring system in future research. Finally, we only evaluated the clinical value of the factors from the 3D reconstruction of CT in RPN. The roles of these parameters in predicting the outcome of conventional laparoscopic PN, open PN, or radical nephrectomy remain to be evaluated. In addition, whether these parameters can help choose optimal surgical modalities also needs to be investigated.

In conclusion, we identified some new parameters based on 3D reconstruction of enhanced CT. TBA and DTA are independently associated with the WIT of RPN. This provides additional assessment value for the complexity of kidney cancer in RPN over the RENAL score. An updated scoring system can assist urologists in identifying patients who may have prolonged WIT (over 25 min) during surgery.

## Data availability statement

The original contributions presented in the study are included in the article/**Supplementary Material**. Further inquiries can be directed to the corresponding author.

## Ethics statement

The studies involving human participants were reviewed and approved by the Ethics Committee of Tongji Hospital. Written informed consent for participation was not required for this study in accordance with the national legislation and the institutional requirements.

## Author contributions

QL and YZ performed the analysis and interpretation of data. ZLiu proposed the conception and designed the roadmap of this research. SW and JL provided administrative support. XM and ZLi evaluated the image results of the patient. ML, HL, WG, ZH, and ZW reviewed and critically revised the manuscript. All authors contributed to the article and approved the submitted version.
