# Supplementary material for: Identification of predictive factors for outcomes after robot-assisted partial nephrectomy based on three-dimensional reconstruction of preoperative enhanced computerized tomography
Source: Front Oncol. 2023 Feb 28;13:927582. doi: 10.3389/fonc.2023.927582 (PMC10011456; doi:10.3389/fonc.2023.927582)
Supplement: Supplementary file 1 [file DataSheet_1.docx]

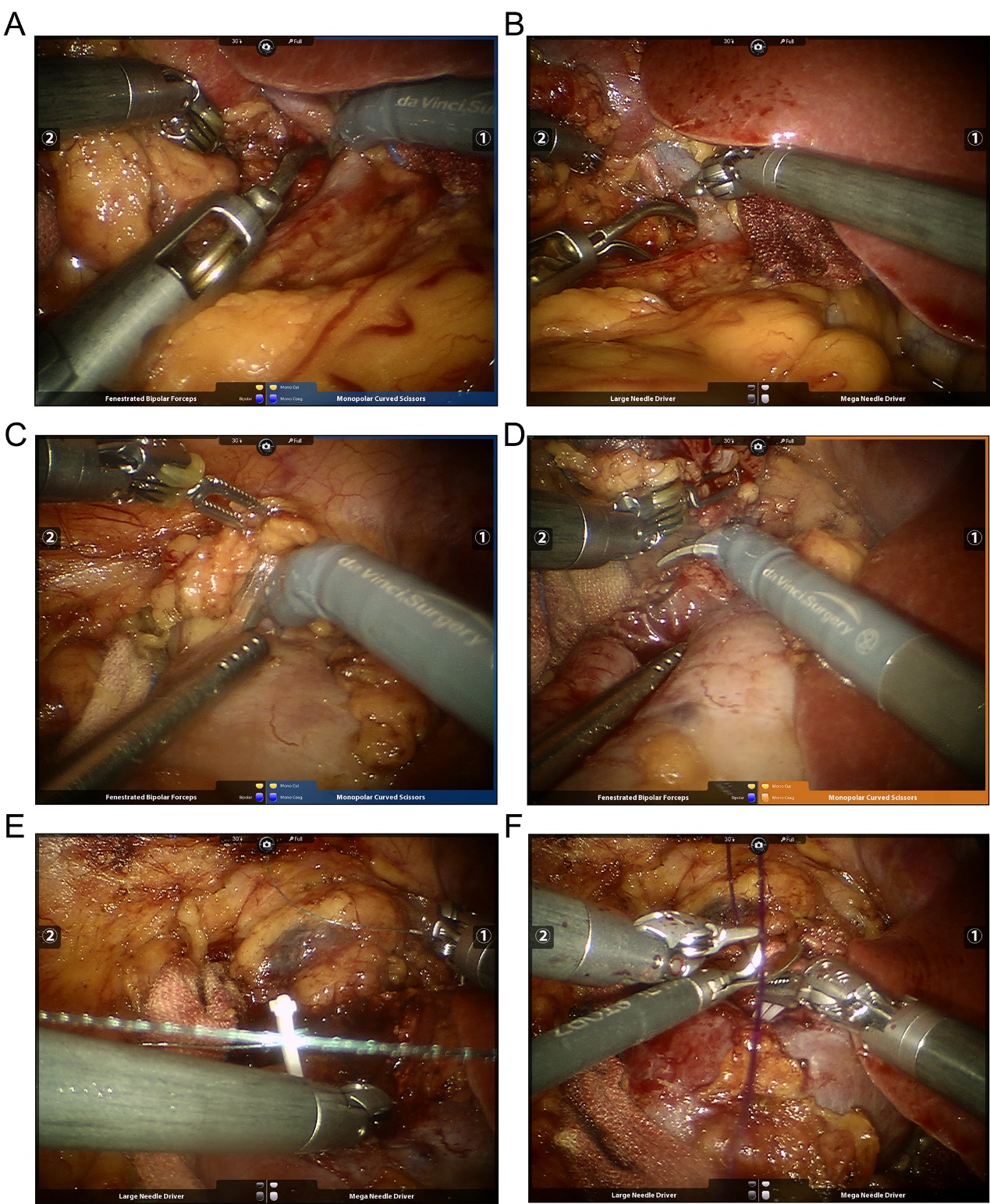


**Figure S1:** Representative images of a surgery record video of a robot-assisted partial nephrectomy. (A) Renal artery clipping; (B) Renal artery reperfusion; (C) The beginning of the incision of renal parenchyma; (D) The end of tumor mass separation; (E) The beginning of the tumor bed suturing; (F) The end of the tumor bed suturing


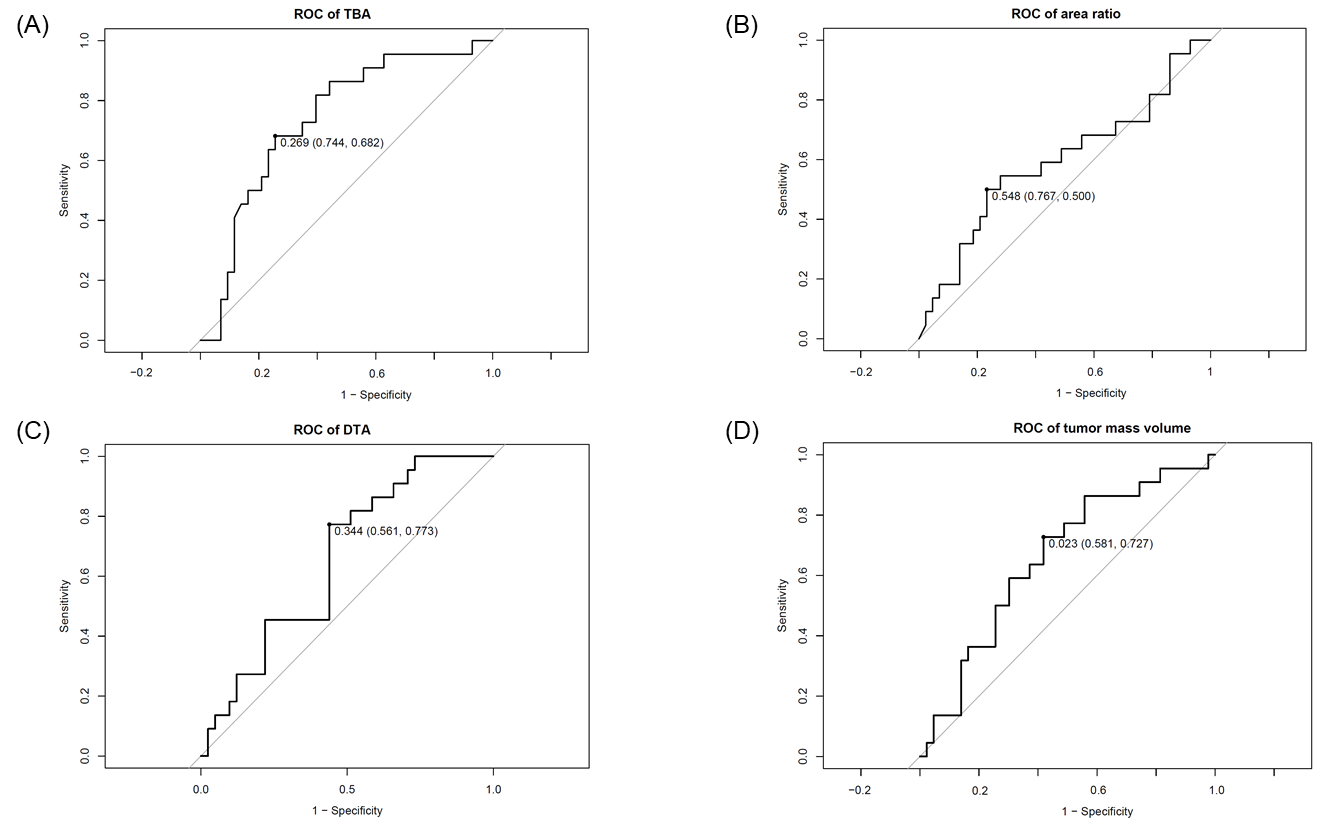


**Figure S2**: The thresholds of tumor bed area, area ratio, distance from tumor to the first bifurcation of renal artery and tumor mass volume


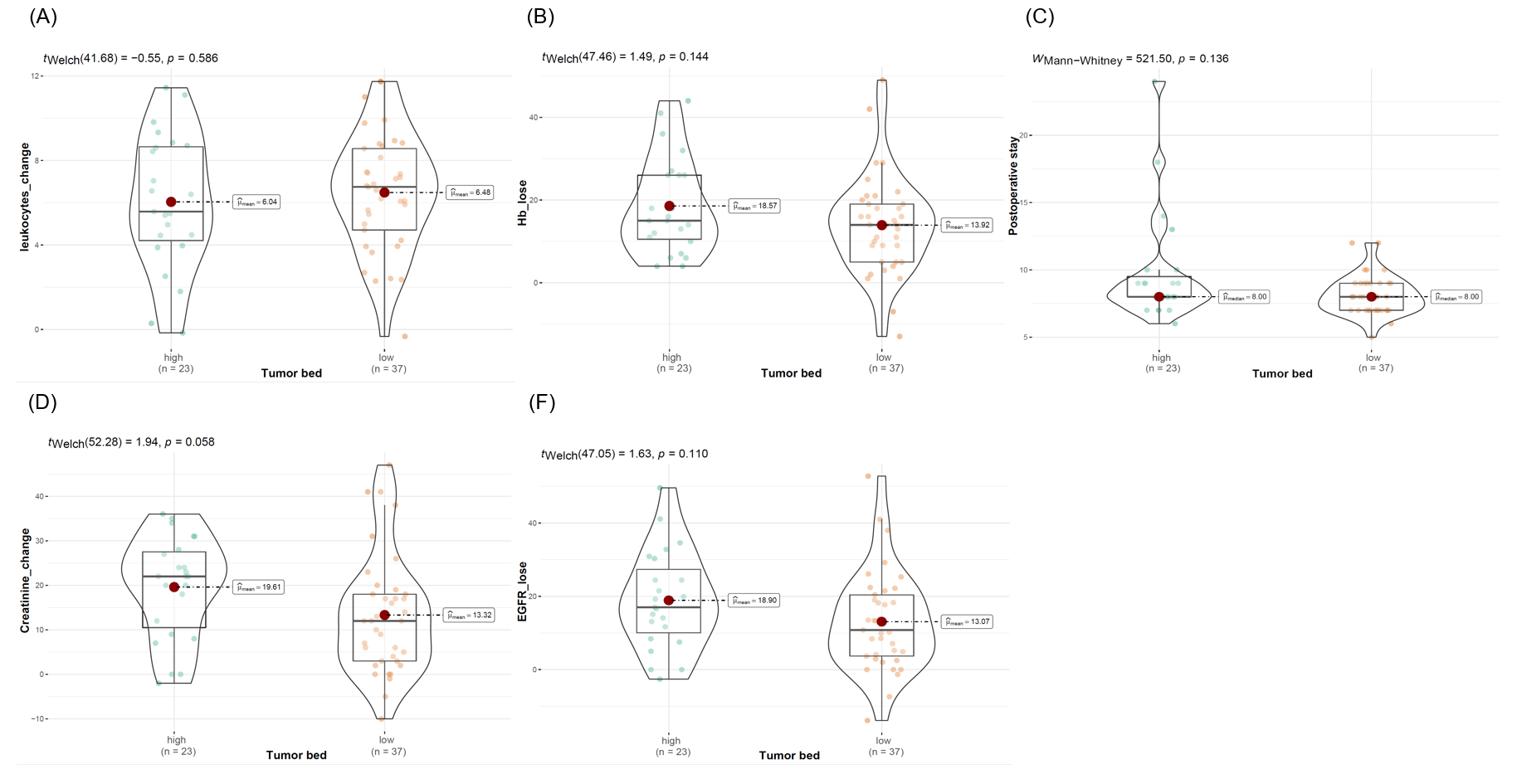


**Figure S3**: Clinical parameters between high- and low- groups of tumor bed area


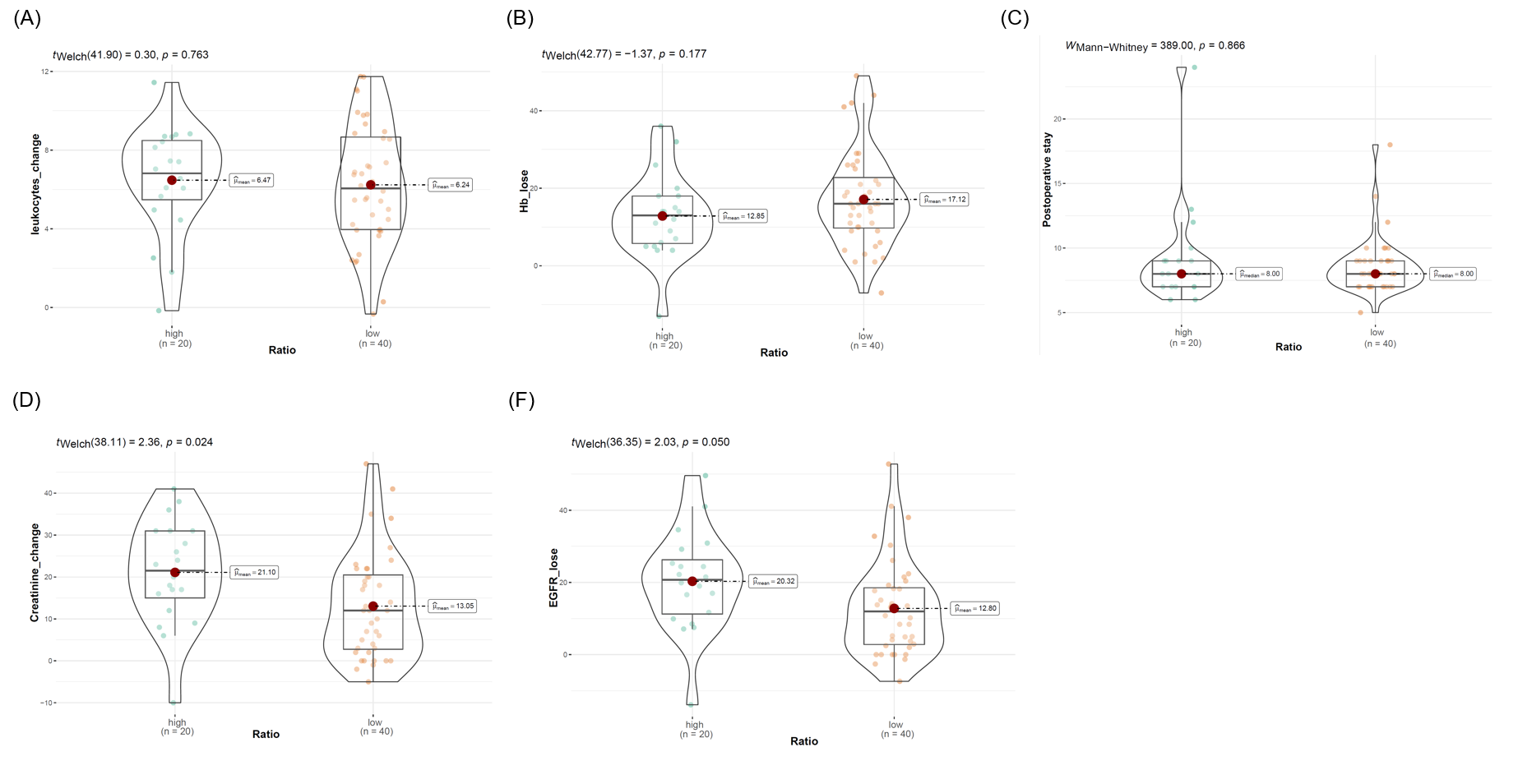


**Figure S4**: Clinical parameters between high- and low- groups of area ratio


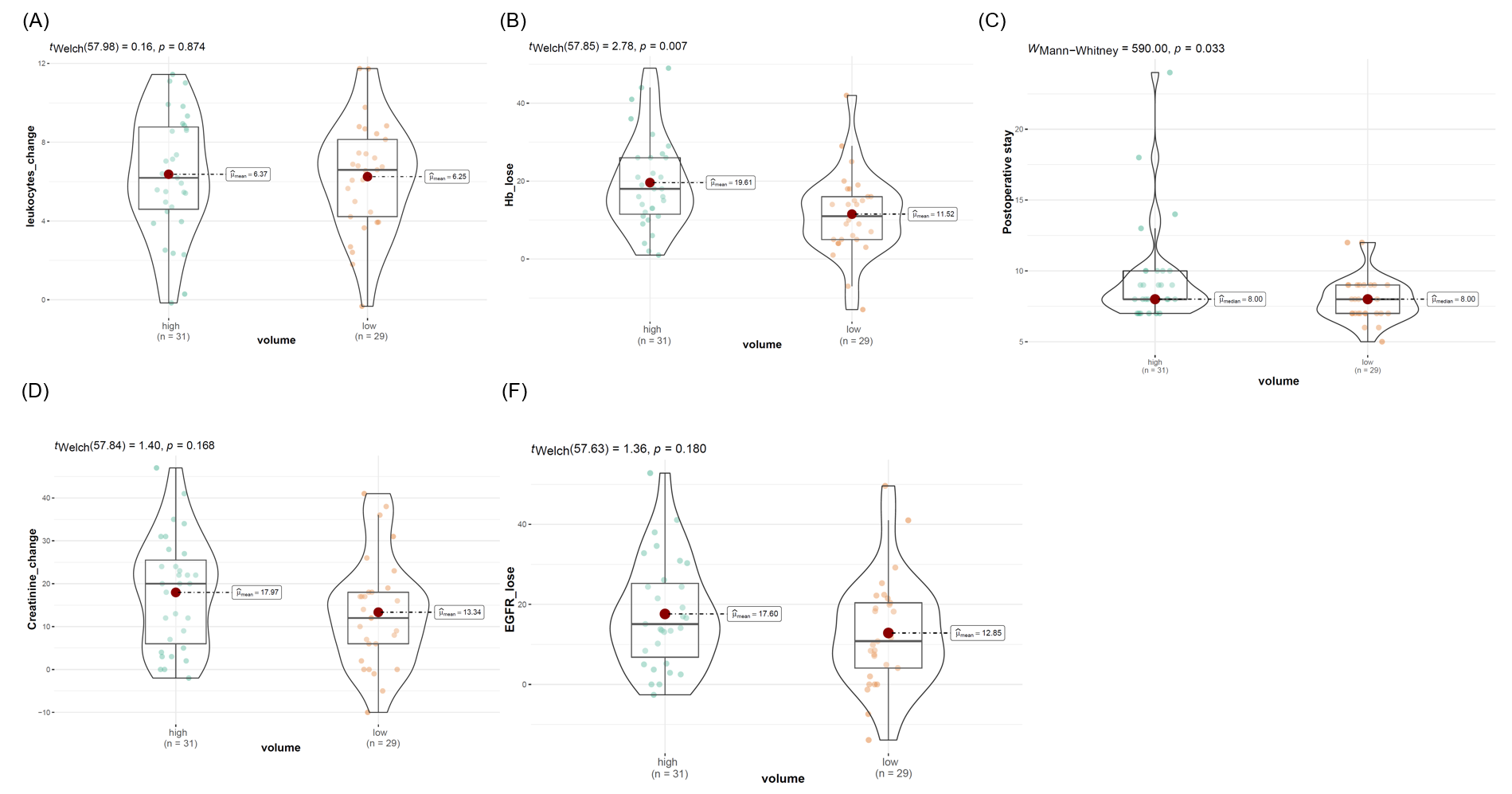


**Figure S5**: Clinical parameters between high- and low- groups of tumor volume


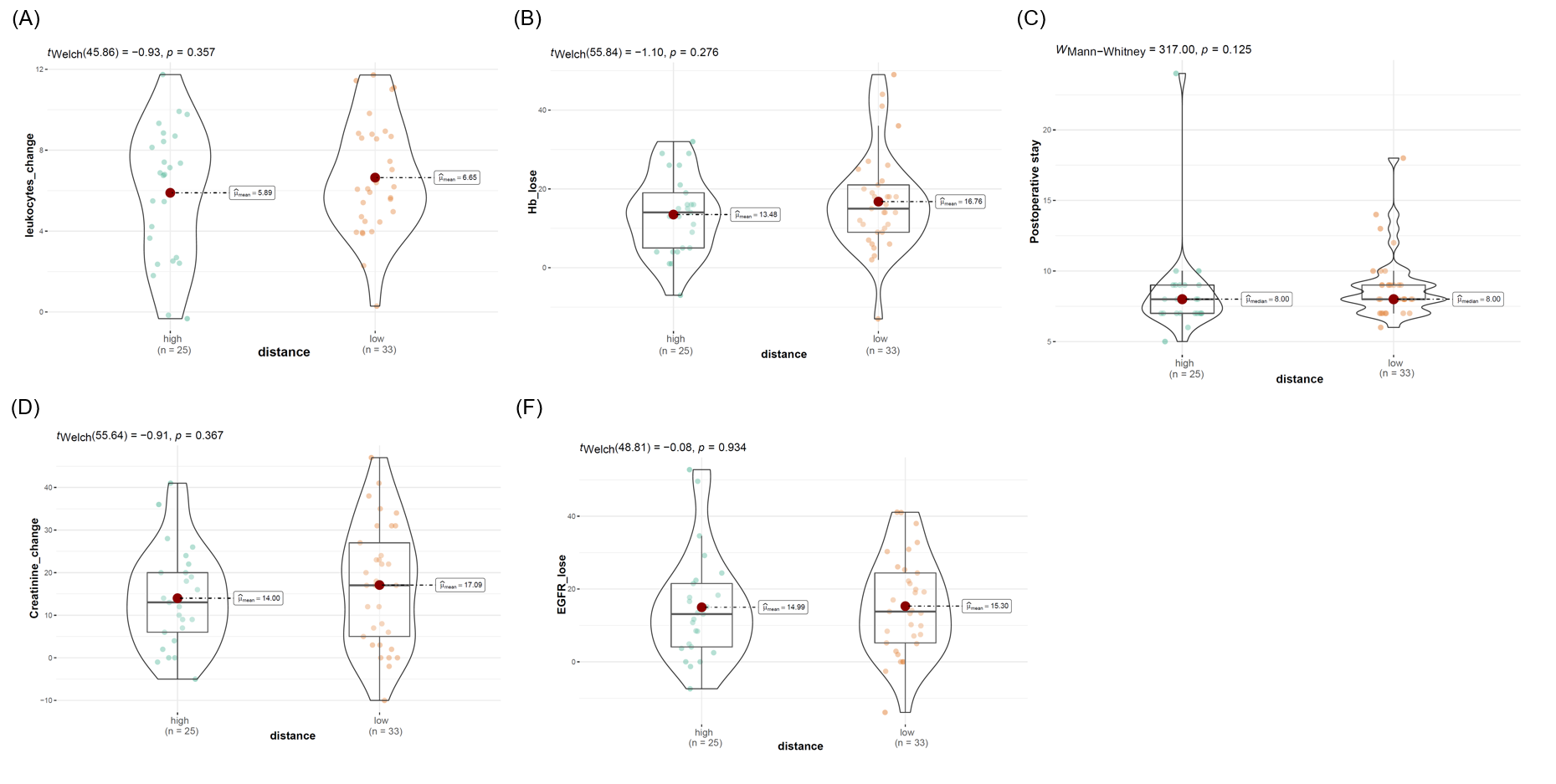


**Figure S6**: Clinical parameters between high- and low- groups of distance from tumor to the first bifurcation of renal artery


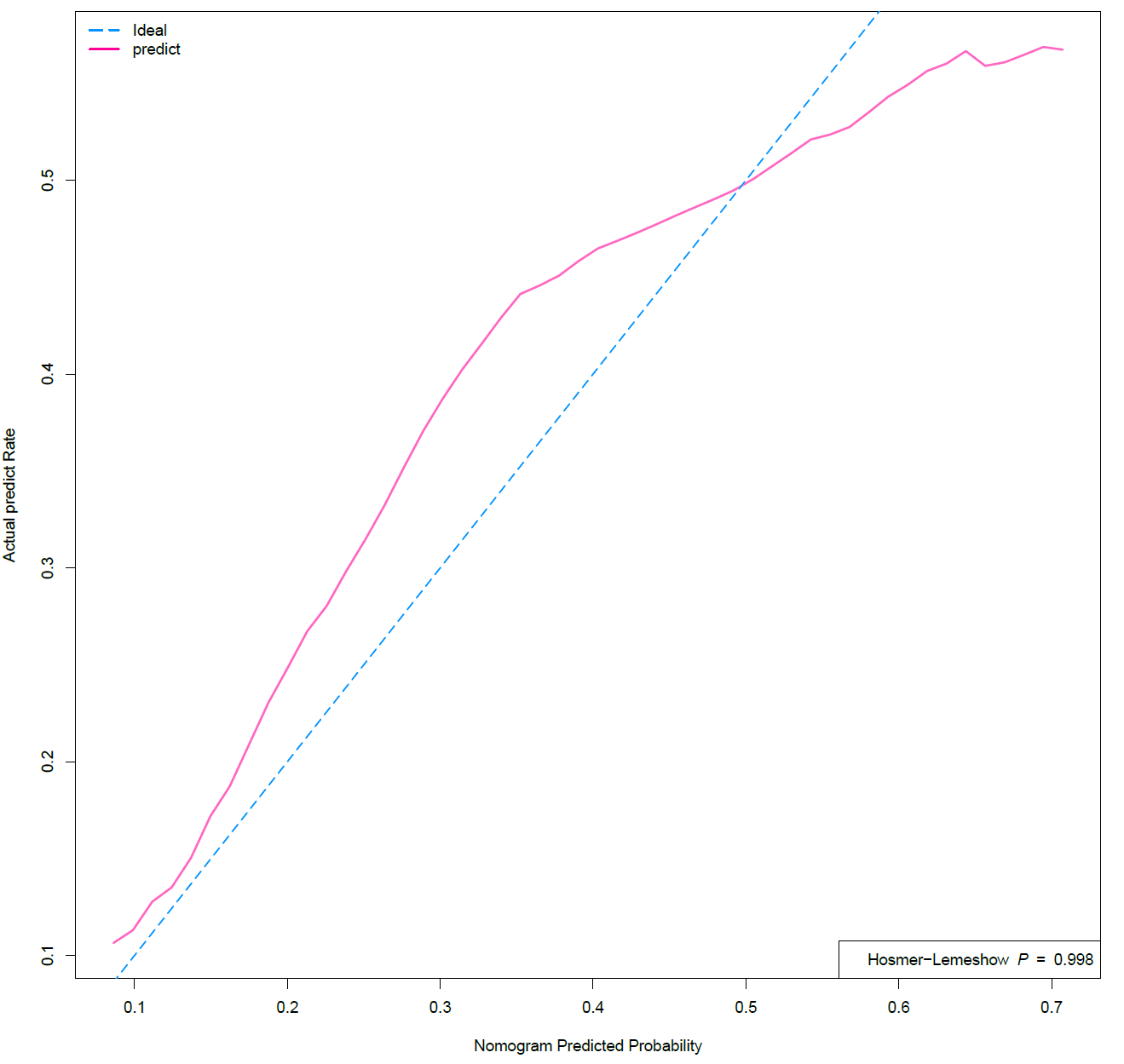


**Figure S7**: The Hosmer–Lemeshow test of the nomogram

**Table S1**: The operative time (minutes) for all patients

| Patient | WIT | TRT | ST | Patient | WIT | TRT | ST | Patient | WIT | TRT | ST |
| --- | --- | --- | --- | --- | --- | --- | --- | --- | --- | --- | --- |
| Patient1 | 39 | 39 | 39 | Patient23 | 24 | 5 | 9 | Patient45 | 19 | 4 | 8 |
| Patient2 | 39 | 18 | 6 | Patient24 | 24 | 7 | 8 | Patient46 | 19 | 6 | 9 |
| Patient3 | 37 | 7 | 11 | Patient25 | 24 | 9 | 5 | Patient47 | 19 | 7 | 9 |
| Patient4 | 36 | 12 | 20 | Patient26 | 24 | 4 | 11 | Patient48 | 19 | 5 | 8 |
| Patient5 | 33 | 14 | 12 | Patient27 | 24 | 6 | 6 | Patient49 | 18 | 9 | 4 |
| Patient6 | 33 | 11 | 6 | Patient28 | 23 | 5 | 6 | Patient50 | 18 | 8 | 5 |
| Patient7 | 31 | 13 | 6 | Patient29 | 23 | 8 | 8 | Patient51 | 16 | 3 | 9 |
| Patient8 | 30 | 8 | 11 | Patient30 | 23 | 10 | 4 | Patient52 | 15 | 4 | 6 |
| Patient9 | 30 | 12 | 8 | Patient31 | 22 | 9 | 8 | Patient53 | 15 | 4 | 5 |
| Patient10 | 30 | 9 | 10 | Patient32 | 22 | 9 | 4 | Patient54 | 15 | 3 | 6 |
| Patient11 | 29 | 8 | 11 | Patient33 | 22 | 5 | 11 | Patient55 | 15 | 3 | 4 |
| Patient12 | 29 | 8 | 7 | Patient34 | 22 | 7 | 7 | Patient56 | 14 | 2 | 5 |
| Patient13 | 27 | 9 | 7 | Patient35 | 21 | 7 | 6 | Patient57 | 14 | 5 | 7 |
| Patient14 | 27 | 6 | 8 | Patient36 | 21 | 4 | 10 | Patient58 | 14 | 5 | 5 |
| Patient15 | 26 | 6 | 8 | Patient37 | 21 | 3 | 7 | Patient59 | 13 | 7 | 5 |
| Patient16 | 26 | 7 | 10 | Patient38 | 21 | 5 | 6 | Patient60 | 11 | 5 | 5 |
| Patient17 | 26 | 9 | 12 | Patient39 | 21 | 8 | 6 | Patient61 | 11 | 5 | 5 |
| Patient18 | 26 | 6 | 10 | Patient40 | 21 | 6 | 5 | Patient62 | 10 | 3 | 5 |
| Patient19 | 25 | 9 | 12 | Patient41 | 20 | 8 | 6 | Patient63 | 10 | 3 | 6 |
| Patient20 | 25 | 9 | 8 | Patient42 | 20 | 7 | 4 | Patient64 | 8 | 3 | 4 |
| Patient21 | 25 | 11 | 15 | Patient43 | 20 | 6 | 10 | Patient65 | 8 | 3 | 4 |
| Patient22 | 25 | 7 | 9 | Patient44 | 19 | 5 | 4 |  |  |  |  |

WIT: warm ischemia time; TRT: tumor resection time; ST: suturing time

**Table S2**. Multicollinearity of the three variables in nom model

|  | Tumor bed | distance | RENAL score |
| --- | --- | --- | --- |
| Multicollinearity | 1.2423 | 1.0455 | 1.2642 |
